# Development of Ring-Expansion RAFT Polymerization of *tert*-Butyl Acrylate with a Cyclic Trithiocarbonate Derivative toward the Facile Synthesis of Cyclic Polymers

**DOI:** 10.3390/molecules29081839

**Published:** 2024-04-18

**Authors:** Jin Motoyanagi, Hiroki Fujii, Masahiko Minoda

**Affiliations:** Faculty of Molecular Chemistry and Engineering, Graduate School of Science and Technology, Kyoto Institute of Technology, Matsugasaki, Sakyo-ku, Kyoto 606-8585, Japan

**Keywords:** RAFT polymerization, cyclic polymer, ring-expansion RAFT polymerization, cyclic trithiocarbonate derivative, *tert*-butyl acrylate

## Abstract

Polymers with cyclic topology have no terminal structure and, therefore, exhibit various unique physical and functional properties compared to those of linear analogs. In this paper, we report an innovative methodology for the synthesis of cyclic polymers via ring-expansion RAFT (RE-RAFT) polymerization of vinyl monomers using a cyclic trithiocarbonate derivative (CTTC) as a RAFT agent. RE-RAFT of *tert*-butyl acrylate (TBA) was performed to yield a mixture of polymers exhibiting a bimodal size exclusion chromatography (SEC) trace. Both the peak top molecular weights shifted to higher-molecular-weight regions as the monomer conversion increased. The structure of the resulting polymer mixture was examined by ^1^H NMR and MALDI-TOF-MS. Detailed studies indicated that the obtained polymer of higher molecular weight was one of the large-sized cyclic polymers generated by the fusion of smaller-sized cyclic polymers during the RE-RAFT polymerization process. This approach opens the door to the simple synthesis of well-controlled cyclic polymers with complex structures, such as alternating and multi-block repeat unit sequences.

## 1. Introduction

In recent years, cyclic polymers with no chain ends have been attracting attention from polymer chemists because of their various unique physical properties, different from those of their corresponding linear counterparts, that is, exhibiting distinguished glass transition temperature, viscosity, diffusion behavior and hydrodynamic volume [1,2,3,4,5]. In addition, it has been reported that linear and cyclic poly(*N*-isopropylacrylamide) (PNIPAMs) show different aggregation behaviors [6,7,8]. PNIPAM is a representative thermo-responsive polymer and has been well-recognized as a promising material in biomedical and biotechnological fields due to its phase transition temperature close to the human body temperature. Furthermore, Tezuka et al. reported that the thermal stability of a self-assembled micelle was significantly affected by a topological factor [9,10,11]. Both the designed amphiphilic, linear triblock copolymer and its cyclized product were self-assembled to form flower-like micelles. Despite no distinctive change in the chemical composition, the cloud point (T_c_) of the cyclic polymer was elevated by more than 40 °C compared to that of its linear counterpart.

As interest in cyclic polymers has increased, many researchers have tried to synthesize these over the past decades. The most straightforward synthesis method is the introduction of reactive sites at both ends of a linear polymer and connecting them to each other via intramolecular coupling; however, this method requires highly diluted conditions to prevent the generation of linear by-products due to intermolecular reactions [3]. Therefore, this method lacks productivity and versatility. On the other hand, ring-expansion polymerization (REP) has been developed as an alternative approach to obtain cyclic polymers. REP is simpler and more mass-productive compared to the above-mentioned conventional method [12]. In REP, by using a cyclic initiator potentially generating a couple of active sites in one molecule, the cyclic topology is maintained by the continuous insertion of monomers throughout the polymerization process. Since the discovery of REP, based on ring-opening metathesis polymerization proposed by Grubbs et al. [13], several new approaches such as coordination polymerization [14] and ionic polymerization [15,16] have been reported to date. However, though the REP so far reported makes it possible to achieve easy access to cyclic polymers, the types of applicable monomers are limited.

**Scheme 1:**
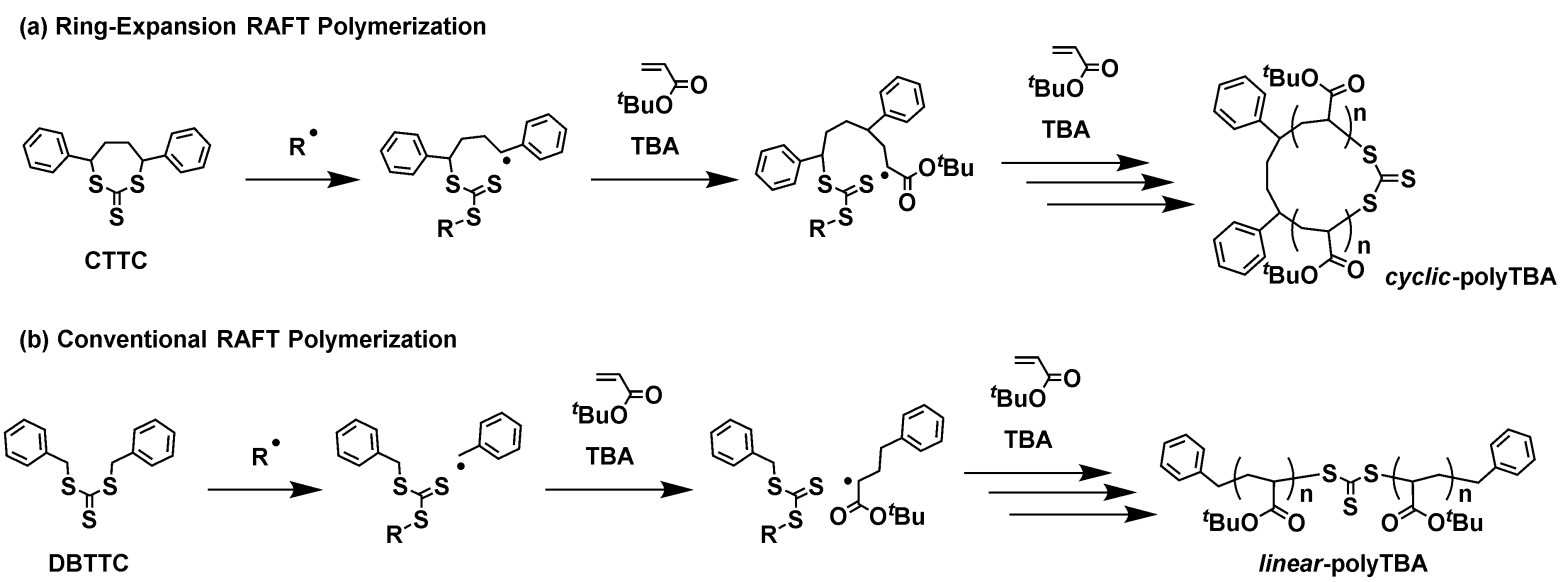
RAFT polymerization of TBA (**a**) with CTTC as a cyclic RAFT agent and (**b**) with DBTTC as an acyclic RAFT agent.

To establish a more versatile synthesis strategy for REP, we focused on reversible addition–fragmentation chain transfer (RAFT) polymerization [17,18,19,20,21,22], which is applicable to a wide range of vinyl monomers such as acrylate, methacrylate and styrene derivatives and has the potential to control the molecular weight and repeating unit arrangement. In addition, a RAFT polymerization system requires no metal catalyst, so it has a low impact on the environment and enables a wide range of applications, including polymer synthesis for biomedical and electronic materials. In this work, we investigated ring-expansion RAFT (RE-RAFT) polymerization conducted with a cyclic trithiocarbonate (4,7-diphenyl-[1,3]dithiepane-2-thione, CTTC) as the RAFT agent (Scheme 1a). According to the previous publications reported by Wang et al. [23] and Lee et al. [24], CTTC was synthesized in a three-step reaction from the commercially available reagents. Wang et al. reported that multi-block polymers linked with trithiocarbonate moieties can be produced by the combination of the ring-opening reaction of CTTC and RAFT polymerization [23].

In this article, we focused on the possibility that CTTC generates two active growth sites within the cyclic molecule, and investigated ring-expansion polymerization of *tert*-butyl acrylate (TBA) using CTTC to synthesize cyclic polymers. Furthermore, to compare its polymerization behaviors as a bifunctional RAFT reagent, we examined the polymerization of TBA using *S*,*S*’-dibenzyl trithiocarbonate (DBTTC), an acyclic RAFT reagent (Scheme 1b). The obtained polymers were characterized in detail by NMR, SEC and MALDI-TOF-MS.

## 2. Results

### 2.1. Ring-Expansion RAFT Polymerization of TBA with CTTC

**Figure 1:**
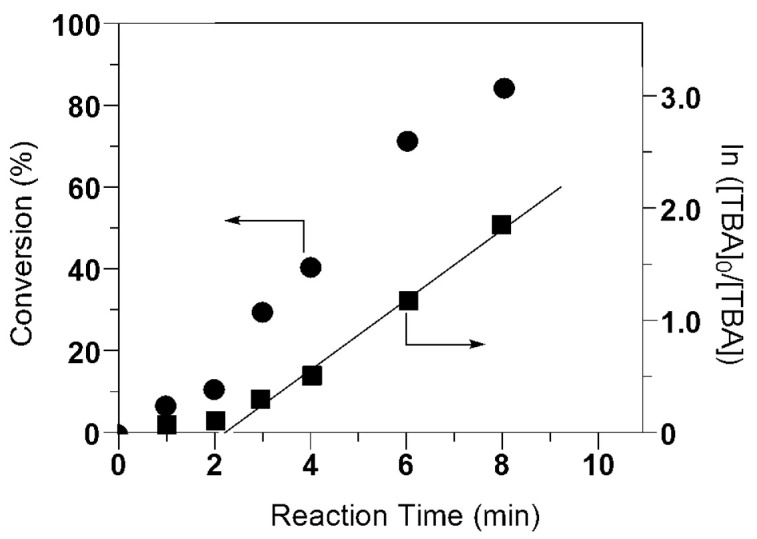
A time-conversion curve (circle) and first-order kinetic plot (square) for the polymerization of TBA. Polymerization was conducted with CTTC/AIBN in anisole at 60 °C ([TBA]_0_/[CTTC]_0_/[AIBN]_0_ = 100/1/0.2, [TBA]_0_ = 20 wt%).

**Figure 2:**
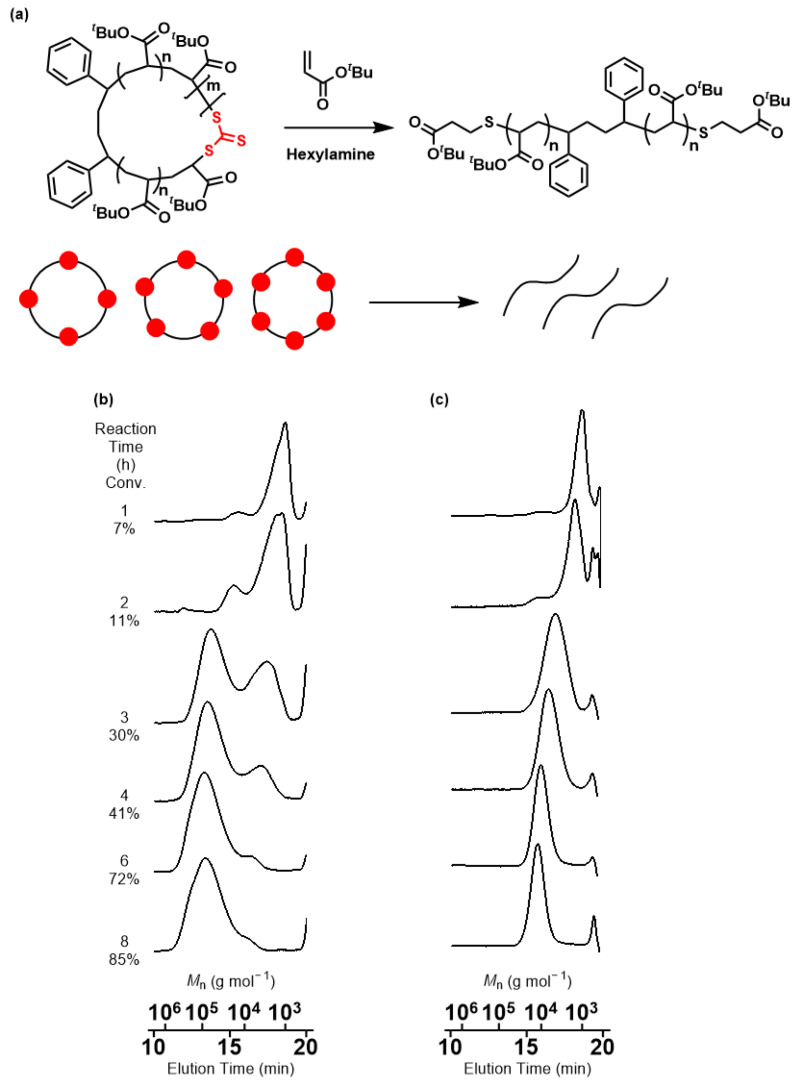
(**a**) Schematic representation of cleaving the segment of polyTBA from the generated polymers by RE-RAFT polymerization of TBA with CTTC (Trithiocarbonate groups are shown in red fonts and red circles). (**b**) SEC curves of polyTBAs measured in THF. Polymerization was conducted with CTTC/AIBN in anisole at 60 °C ([TBA]_0_/[CTTC]_0_/[AIBN]_0_ = 100/1/0.2, [TBA]_0_ = 20 wt%). (**c**) SEC curves of cleaving the segment of polyTBA from the generated polymers by RE-RAFT polymerization of TBA with CTTC obtained at various TBA conversions.

**Scheme 2:**
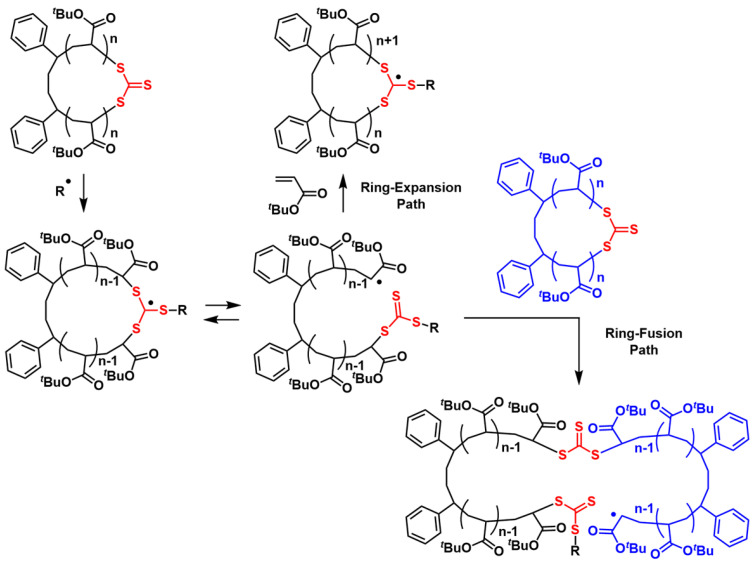
Schematic of ring-expansion RAFT polymerization of TBA with a cyclic RAFT reagent CTTC containing “ring-expansion paths” and “ring-fusion paths”.

We performed the RAFT polymerization of TBA using CTTC as a cyclic RAFT reagent in anisole at 60 °C ([TBA]_0_/[CTTC]_0_/[AIBN]_0_ = 100/1/0.2, [TBA]_0_ = 20 wt%). In Figure 1, the time-conversion curve obtained for the polymerization of TBA shows an induction phase of about 2 h, and that we reached over 85% conversion within 8 h. After the induction phase, the first-order kinetic plot for the polymerization of TBA exhibits a linear relationship, indicating that the concentration of the growing active species remained constant throughout polymerization, thus supported the notion that a controlled polymerization process occurred. However, SEC analysis of the polymers produced showed that the polymerization behavior needed to be reconsidered, that is, an almost unimodal chromatogram was observed at the very early stage of polymerization (Conv. ~10%), whereas after the middle stage of polymerization, the chromatograms showed bimodal peaks, including a new peak in the higher-molecular-weight region (Figure 2a). This phenomenon was due to the simultaneous occurrence of two paths in the present ring-expansion polymerization. One was the “ring-expansion path”, leading to the synthesis of cyclic polymers via ring-expansion polymerization, which occurred by monomer insertion into the cyclic species, and the other was the “ring-fusion path” due to the intermolecular chain transfer reactions between the cyclic polymers formed, containing one or more trithiocarbonate moieties. The latter path resulted in generating higher-molecular-weight cyclic polymers (Scheme 2) [16]. To suppress the occurrence of the “ring-fusion path”, we attempted ring-expansion polymerizations with varied initial monomer concentrations. Although the monomer concentration was decreased from 20 wt% to 10 wt% and 5 wt%, the appearance of the higher-molecular-weight polymers due to ring fusion was still observed. When the monomer concentration was further reduced to 1 wt%, the radical concentration became too low, and no polymerization occurred. Considering the reaction mechanism of the ring fusion, the obtained high-molecular-weight polymers can be thought to possess a cyclic structure, where multiple polyTBA segments are connected to each other through trithiocarbonate (TC) moieties. Therefore, cleavage of the TC moieties can afford individual polyTBA segments. Detailed investigation of the obtained polyTBA segment polymers can afford useful information about the polymerization behaviors of RE-RAFT polymerization. It is known that the TC group is hydrolyzed by adding primary amines to give a thiol group so that telechelic polyTBAs with thiol functions at both ends will be formed. However, under basic conditions, the terminal thiol groups easily form inter- or intramolecular disulfide bonds. To avoid disulfide formation, an excess amount of TBA was added after hydrolysis, and then the thiol groups generated were capped with the acryloyl group of TBA by a Michael reaction. We carried out hydrolysis of the polymers obtained by RE-RAFT polymerization via treatment with hexylamine and then TBA in THF. After reaction for 3 h at room temperature, the UV-vis spectrum of the reaction mixture showed disappearance of the 310 nm peak assignable to the TC moiety, supporting the notion that quantitative hydrolysis proceeded. The polyTBAs obtained by the cleavage reaction were then characterized by SEC to examine the growth of the polyTBA segments during RE-RAFT polymerization (Figure 2b, Table 1). Irrespective of the reaction time, the SEC curves exhibited a unimodal peak, and the peak top shifted to higher-molecular-weight regions as the monomer conversion increased, while keeping molecular weight distributions of ca. 1.4 or below. Furthermore, it is notable that the *M*_n_s of the polyTBA segment polymers agreed with the theoretical molecular weights calculated from the [TBA]_0_/[CTTC]_0_ feed ratios and monomer conversions. These results revealed that the polymerization of TBA in RE-RAFT polymerization with a cyclic RAFT reagent, CTTC, proceeded in a controlled manner.

### 2.2. Characterization of the Polymers Obtained by RE-RAFT Polymerization with CTTC

**Figure 3:**
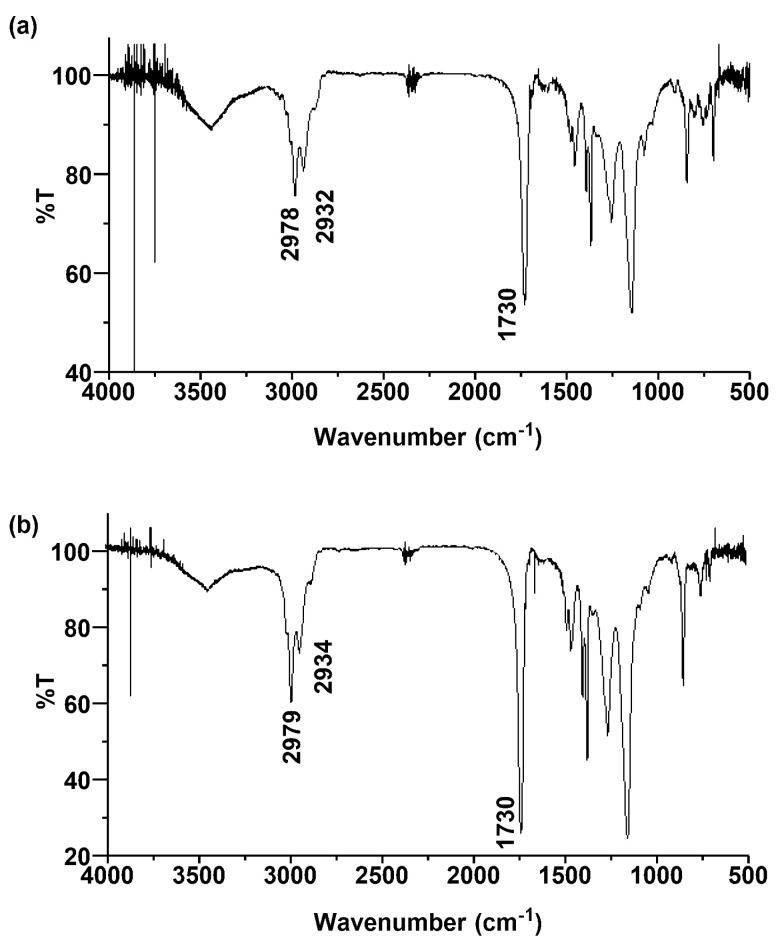
FT-IR spectra of (**a**) cyclic polyTBA synthesized by CTTC/AIBN and (**b**) linear polyTBA synthesized by DBTTC/AIBN using a KBr pellet.

**Figure 4:**
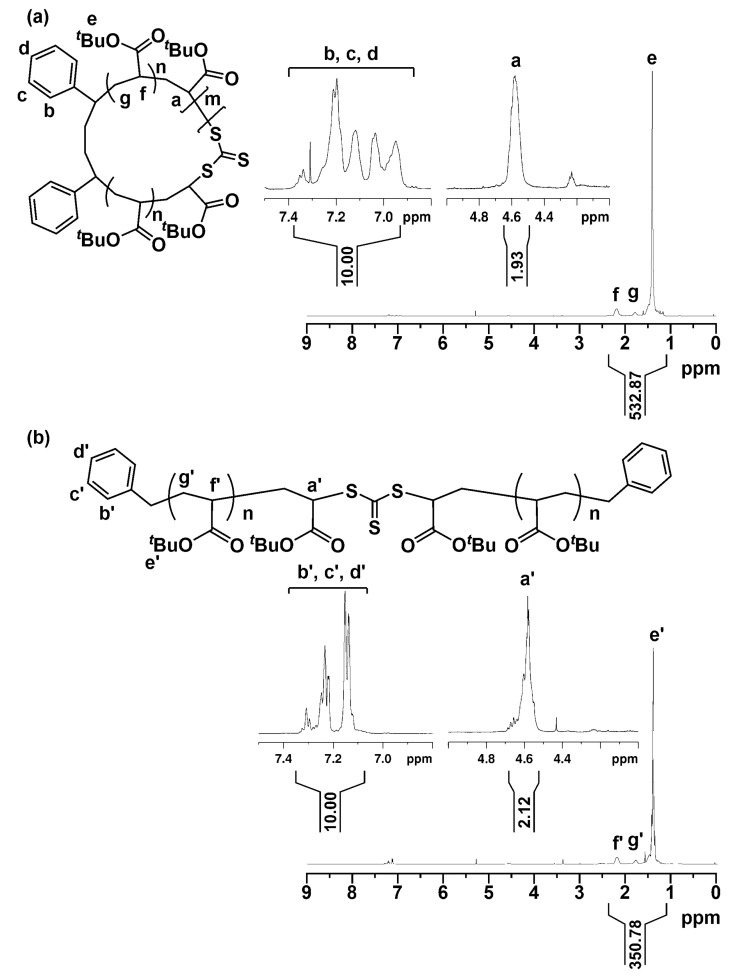
^1^H NMR spectra of (**a**) cyclic polyTBA synthesized by CTTC/AIBN and (**b**) linear polyTBA synthesized by DBTTC/AIBN in CD_2_Cl_2_.

After removal of the unreacted TBA by preparative SEC, the structure of the isolated polyTBA (Conv. of TBA = 43%) was analyzed in detail by FT-IR and ^1^H NMR spectroscopy (Figure 3a). As a reference polymer, a linear polyTBA was separately synthesized by conventional RAFT polymerization with DBTTC as an acyclic RAFT reagent in anisole at 60 °C ([TBA]_0_/[DBTTC]_0_/[AIBN]_0_ = 100/1/0.2, [TBA]_0_ = 20 wt%, Conv. of TBA = 31%; linear polyTBA: *M*_n_ = 4100, *M*_w_/*M*_n_ = 1.20) (Scheme 1). The FT-IR spectroscopy of cyclic polyTBA and linear polyTBA using KBr pellets showed similar spectra (Figure 3). C-H and C=O stretching vibrations were confirmed at 2979 cm^−1^ and 1730 cm^−1^, respectively. The ^1^H NMR spectrum of the linear polyTBA is depicted in Figure 4b. The phenyl protons (peaks b’, c’ and d’) arising from the termini of the linear polyTBA appeared at 7.1–7.3 ppm, and those of the pair of methine protons (peak a’) adjacent to the TC moiety appeared at 4.6 ppm. The peak intensity ratio of the phenyl protons and methine protons was determined to be 10.0:2.12, which was in good agreement with the expected telechelic structure (Figure 4b). The number-average degree of polymerization (*DP*_n_) of the linear polyTBA was evaluated to be 29 by the peak intensity ratio of the *tert*-butyl protons (peak e’) and the terminal phenyl protons (peaks b’, c’ and d’). This *DP*_n_ value was in good agreement with the theoretical one calculated based on the feed molar ratio of TBA to DBTTC and the monomer conversion. The chemical structure of the resulting polyTBA based on the reaction mechanism of the RE-RAFT polymerization is shown in Figure 4a. In the ^1^HNMR spectrum of the polyTBA obtained by RE-RAFT polymerization, phenyl proton peaks b, c and d derived from the CTTC residue appeared at 6.9–7.4 ppm and the characteristic peak a at 4.6 ppm of the methine protons adjacent to the TC moiety was also observed. The ratio of the resonance intensities of the phenyl protons versus methine protons [Figure 4a, peaks b–d and a, respectively] was 10.0:1.93, which indicated that TBA monomers were inserted between the C-S bond of the TC moiety and the benzyl carbon (-S-C(=S)-*S*-*C*H(Ph)CH_2_CH_2_-) in the initiation process of RE-RAFT polymerization and consecutive insertions during propagation. These results clearly indicated that the molecular structure thoroughly retained the cyclic structure of CTTC and that the resulting polyTBAs consisted of a cyclic structure without termini. Furthermore, the *DP*_n_ of the polyTBA segments was evaluated to be 44 from the intensity ratio of the peaks of the CTTC moiety (peaks b, c and d) and pendant *tert*-butyl protons. This *DP*_n_ value was nearly identical to the theoretical one calculated based on the [TBA]_0_/[CTTC]_0_ feed ratio and the monomer conversion. These data strongly supported the notion that RAFT polymerization of TBA with CTTC as a cyclic RAFT reagent proceeded in the ring-expansion polymerization process, and thus the present system was confirmed to constitute RE-RAFT polymerization.

**Figure 5:**
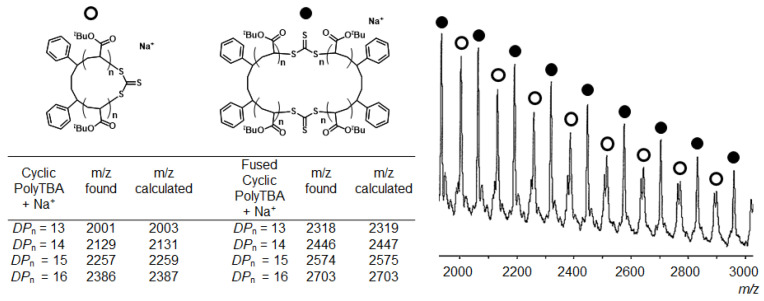
MALDI-TOF-MS spectrum of the cyclic polyTBA (matrix: 9-nitroanthracene; cationizing agent: sodium trifluoroacetate).

To provide further evidence that the polymers obtained with CTTC possessed a cyclic structure, we performed MALDI-TOF-MS measurements (matrix: 9-nitroanthracene; cationizing agent: sodium trifluoroacetate). To obtain data with better resolution, we employed a polymer sample produced at the early stage of RE-RAFT polymerization. Figure 5 (right) shows the MALDI-TOF-MS spectrum of the polyTBA synthesized by RE-RAFT polymerization at Conv. = 12%, where two different sets of peaks can be clearly observed. The peak group indicated by black circles (peaks ●) was assigned to the fused-cyclic polyTBAs, whereas the group shown by white circles (peaks ○) was assigned to the non-fused monomeric-cyclic polyTBAs, with one CTTC residue in the circle. The structures of these cyclic polyTBAs are shown in Figure 5 (left). In addition, observed and calculated *m*/*z* values for the non-fused and fused cyclic polyTBAs with different *DP*_n_ values are summarized in Figure 5, in which the calculated *m*/*z* values correspond to those of [CTTC-(TBA)n + Na] + (*M*_n_ = 316 + (128 × n) + 23). The observed and calculated *m/z* values are nearly identical, which means that the obtained polyTBAs had no termini structure, and this provides strong support for the notion that the polyTBAs obtained were composed only of a cyclic structure.

## 3. Materials and Methods

### 3.1. Chemicals and Reagents

Unless otherwise stated, all commercial reagents including 2,2′-Azobis(isobutyronitrile) (AIBN; FUJIFILM Wako Pure Chemical Corporation, Osaka, Japan, 98%) were used as received. *tert*-Butyl acrylate (TBA; FUJIFILM Wako Pure Chemical Corporation, Osaka, Japan, 97%) was distilled from CaH_2_ under reduced pressure. A cyclic trithiocarbonate derivative, 4,7-diphenyl-[1,3]dithiepane-2-thione (CTTC), was synthesized according to the literature [23,24].

### 3.2. Methods

^1^H NMR spectra were assessed in CDCl_3_ or CD_2_Cl_2_ at 25 °C on a Bruker model AC-500 spectrometer (Bruker, Billerica, MA, USA), operating at 500 MHz, where chemical shifts (*δ* in ppm) were determined with respect to C*H*Cl_3_ or C*H*_2_Cl_2_ as the internal standard, respectively. Analytical size exclusion chromatography (SEC) was performed at 40 °C, using 8.0 mm × 300 mm polystyrene gel columns (TOSOH-TSKgel H type × 2) on a TOSOH GPC-8320 system, equipped with a UV-8000 variable-wavelength UV-vis detector (TOSOH, Tokyo, Japan). The number-average molecular weight (*M*_n_) and dispersity ratio (*M*_w_/*M*_n_) were calculated from the chromatographs with respect to 15 polystyrene standards (Scientific Polymer Products, Inc., Ontario, NY, USA; *M*_n_ = 580–670,000 g mol^−1^, *M*_w_/*M*_n_ = 1.01–1.07). Preparative size exclusion chromatography (SEC) was performed at 25 °C by using 21.5 mm × 300 mm polystyrene gel columns (TOSOH TSKgel G2000H, G2500H and G3000H) on a TOSOH model CCPE equipped with a RI-8022 RI detector (TOSOH, Tokyo, Japan). Matrix-assisted laser desorption/ionization–time-of-flight mass spectrometry (MALDI-TOF-MS) was performed on a Bruker model amaZon SL spectrometer (Bruker, Billerica, MA, USA) using THF as a solvent, 9-nitroanthracene as a matrix and sodium trifluoroacetate as a cationizing agent. UV-vis spectra were recorded using a quartz cell of 1 cm path length on a SHIMADZU Type UV-2550 spectrometer (SHIMADZU, Kyoto, Japan). Infrared spectra were recorded at 25 °C on a JASCO model FT/IR-610 Plus Fourier transform infrared spectrometer (JASCO, Hachioji, Japan).

### 3.3. Ring-Expansion RAFT Polymerization of TBA with CTTC

Ring-expansion RAFT polymerization of TBA was carried out with CTTC as a cyclic RAFT agent and AIBN as an initiator. To a solution of TBA (320 mg, 2.5 mmol) and CTTC (7.9 mg, 25 μmol) in anisole (1.30 g), we added AIBN (0.82 mg, 5.0 μmol) in a glass tube ([TBA]_0_/[CTTC]_0_/[AIBN]_0_ = 100/1/0.2). The resulting solution was degassed by three freeze–pump–thaw cycles, and then the glass tube was sealed under vacuum, heated at 60 °C for 1–8 h and quenched by rapid cooling. The reaction mixture was analyzed by SEC and ^1^H NMR spectroscopy. The anisole solution of the reaction mixture was poured into a large amount of MeOH (30 mL) to precipitate the polymers and remove the unreacted monomers. The resultant polymer was collected by centrifugation and dried under reduced pressure. The residue was subjected to preparative SEC, where the first fraction was collected and evaporated to dryness to give the target polymer. The isolated polymer structure was analyzed by ^1^H NMR spectroscopy and MALDI-TOF-MS spectroscopy.

### 3.4. Conventional RAFT Polymerization of TBA with DBTTC

Conventional RAFT polymerization of TBA was carried out with DBTTC as a bifunctional acyclic RAFT agent and AIBN as an initiator. To a solution of TBA (320 mg, 2.5 mmol) and DBTTC (7.3 mg, 25 μmol) in anisole (1.30 g), we added AIBN (0.82 mg, 5.0 μmol) in a glass tube ([TBA]_0_/[DBTTC]_0_/[AIBN]_0_ = 100/1/0.2). The resulting solution was degassed by three freeze–pump–thaw cycles, and then the glass tube was sealed under vacuum, heated at 60 °C for 1–8 h and quenched by rapid cooling. The reaction mixture was then treated in the same way as above.

## 4. Conclusions

In this study, we demonstrated a new methodology for the simple and efficient synthesis of cyclic polymers via ring-expansion RAFT (RE-RAFT) polymerization of an acrylate monomer using a cyclic RAFT reagent, CTTC. The RE-RAFT of TBA resulted in a mixture of polymers with a bimodal SEC trace. The structure of the resulting polymers was analyzed using ^1^H NMR and MALDI-TOF-MS. It was found that the polymer with a higher molecular weight was a large cyclic polymer formed by the fusion of smaller cyclic polymers during the RE-RAFT polymerization process. To the best of our knowledge, this study is the first to successfully synthesize cyclic polyacrylates by radical polymerization using a cyclic RAFT agent. The present RE-RAFT polymerization is indeed accompanied by “ring-fusion” reactions during polymerization; however, the polymer chains grow while retaining the cyclic structure, allowing easy and mass-productive synthesis of cyclic polymers. In addition, this approach opens the door to designing welldefined cyclic polymers composed of various monomers and novel cyclic polymers with complex architectures such as multi-block and alternating repeat unit sequences. Large-scale synthesis of cyclic polymers enables us to elucidate the relationships between the physical and functional properties of cyclic polymers and their structures in detail. In our group, the syntheses of stimuli-responsive cyclic polymers, grafted cyclic polymers and cyclic polymers with multi-block and alternating structures are in progress.

## Figures and Tables

**Table 1:** Molecular weights (*M*_n_) and dispersities (*M*_w_/*M*_n_) of the segments of polyTBAs obtained by the cleavage of the polymers generated from RE-RAFT polymerization of TBA with a cyclic RAFT reagent, CTTC.

Reaction Time (h)	TBA Conversion (%) ^a^	*M*_n, theory_ ^b^ (g mol^−1^)	*M*_n, cleaved_ ^c^ (g mol^−1^)	*M*_w_/*M*_n_ ^c^
1	7	900	890	1.12
2	11	1400	1300	1.14
3	30	3800	3800	1.44
4	41	5300	5400	1.33
6	72	9200	9100	1.20
8	85	11,000	11,000	1.15

^a^ Determined by ^1^H NMR. ^b^ Calculated from *M*_n, theory_ = ([TBA]_0_/[CTTC]_0_ × (molecular weight of TBA) × (conversion of TBA). ^c^ Estimated by PSt-calibrated SEC in THF.

## Data Availability

The original contributions presented in the study are included in the article, further inquiries can be directed to the corresponding authors.
